# Determinants of electronic cigarette use among Finnish adults: Results from a population-based survey

**DOI:** 10.1177/1455072517736618

**Published:** 2017-11-21

**Authors:** Otto Ruokolainen, Hanna Ollila, Karoliina Karjalainen

**Affiliations:** National Institute for Health and Welfare, Finland; National Institute for Health and Welfare, Finland; National Institute for Health and Welfare, Finland

**Keywords:** e-cigarette, electronic cigarette, endgame, health policy, nicotine, smoking, snus, tobacco control, tobacco products

## Abstract

**Background:**

Electronic cigarette (e-cigarette) use is rising in the Western world, but studies from the Nordic countries are lacking. Many countries are implementing policy changes, brought about for example by the 2014 European Tobacco Products Directive, and monitoring e-cigarette use is considered important. The aim of this article is to account for the prevalence of e-cigarette use among the Finnish adult population and to examine correlates of ever use and current use of e-cigarettes prior to some changes in the Finnish regulatory scheme.

**Methods:**

A population-based survey was conducted in 2014. A representative random sample (*N* = 7000) of Finnish people aged 15–69 years was drawn from the Finnish Population Information System. Data were collected by self-administered anonymous online/postal questionnaire. The response rate was 50% (*n* = 3485). A multinomial logistic regression model was used to estimate the association between e-cigarette use and different explanatory variables.

**Results:**

Of all participants, 2% were current and 12% were ever users of e-cigarettes. Younger age and current or previous tobacco use increased the odds for both current and ever use of e-cigarettes when compared with never users. Unemployment and lower education were associated with current e-cigarette use and being a student was associated with ever use of e-cigarettes.

**Conclusions:**

The current use of e-cigarettes in the adult population is low in Finland, having at least tried is more common. Both types of e-cigarette use are concentrated to groups considered to be more vulnerable, such as younger people and those with a lower socioeconomic position. Further monitoring of e-cigarette use is needed in view of Finland’s aim to become nicotine and tobacco free by 2030.

Electronic cigarette (e-cigarette) use is rising fast in Western countries (Hajek, Etter, Benowitz, Eissenberg, & McRobbie, 2014; McMillen, Gottlieb, Whitmore Shaefer, Winickoff, & Klein, 2015). According to the Eurobarometer 2014 data, the prevalence of ever use of e-cigarettes was 11.6% at the EU level (Filippidis, Laverty, Gerovasili, & Vardavas, 2017). In the Nordic countries, the prevalence of ever use exceeds this European average in Denmark (15.8%) and in Finland (13.2%), whereas in Sweden (7.9%) the prevalence is lower (Filippidis et al., 2017). The European average for current use of e-cigarettes is 2%, and differences between the Nordic countries are less pronounced than for ever use (European Commission, 2015).

The legal status and policies on e-cigarettes vary between the Nordic countries. According to Kennedy, Awopegba, De León, and Cohen (2016), Denmark and Finland share mostly the same regulatory domains related to e-cigarettes, whereas Norway and Iceland seem to regulate them in fewer domains than Denmark and Finland. Although the prevalence of adult e-cigarette use and its associations with other factors have been reported in many surveys, the results from the Nordic countries with stringent tobacco control are rare (Ruokolainen, Ollila, Sandström, & Heloma, 2016; see also Filippidis et al., 2017; Vardavas, Filippidis, & Agaku, 2015).

Finland has had regulative tobacco control for decades (Patja, 2014), and it has also been one of the strictest regulators of e-cigarettes in Europe. In 2010, Finland was the first country to change the objective of the national Tobacco Control Act (TCA) from reducing tobacco use to ending the use of tobacco products altogether by 2040. In 2016 the TCA was revised, taking effect on 15 August (Finlex, 2016a), whereby the goal was brought forward from 2040 to 2030. The scope of the goal was broadened to include the use of “other nicotine-containing products that are toxic to humans and cause addiction” (Finlex, 2016a). The medicinal use of nicotine is excluded from the scope of the application of the TCA with a restrictive provision. Nicotine replacement therapy continues to be regulated under the Medicines Act (Finlex, 2013).

In Europe, the new EU Tobacco Products Directive (TPD) (Eur-Lex, 2014) took the first steps towards harmonising the regulation of e-cigarettes in the member states. However, the directive left the actual sales of the products to be regulated under national jurisdiction. In Finland, national regulation of e-cigarettes was aligned in the 2016 TCA with tobacco product regulation, which involved retailer licensing, age limits, a point-of-sale display ban, a ban on characterising flavours, and prohibition of online and distance sales of both nicotine-free and nicotine-containing e-cigarettes. The marketing and promotion of e-cigarettes was completely prohibited already before the new Tobacco Control Act. Taxation for both nicotine and non-nicotine liquids was enacted from the beginning of 2017 (Finlex, 2016b).

Prior to the new TCA, nicotine-containing e-cigarettes were regulated as medicinal products for smoking cessation. Hence, a marketing authorisation from the Finnish Medicines Agency (FIMEA) had to be applied, and the products had to meet the same quality, safety, and efficacy requirements as nicotine replacement therapy (NRT) products. No medicinal e-cigarettes had been introduced to the Finnish market by 2017, and nicotine-containing e-cigarettes could only be purchased from international online retailers or imported from travels abroad. As the new TCA allows also the sales of nicotine-containing e-cigarettes and e-liquids in regular stores and kiosks with only a retailer licence, the availability of these products is likely to increase. A marketing authorisation for nicotine-containing e-cigarettes intended for medicinal use can still be applied.

As e-cigarette regulation has now become an integral part of tobacco control policies, we need to gather population-based knowledge on e-cigarette use in order to evaluate the impact of different policies. In addition, profound knowledge about the phenomenon helps to develop and target preventive measures. This article presents results of e-cigarette use and its determinants among Finnish adults in 2014, two years before the e-cigarette legislation changed from regulating them as only medicinal products into regulating e-cigarettes as tobacco-like products. We separate e-cigarette users into ever users and current users and study which factors are associated with e-cigarette use in these groups. We also study the use of nicotine-containing e-liquids in a regulatory environment where they have been available only from abroad, either by distance sales or by personal import.

## Material and methods

### Data

A population-based drug survey concerning drug and other substance use was conducted in 2014. A representative random sample (*N* = 7000) of Finns aged 15–69 years was drawn from the Finnish Population Information System. The Åland Islands, the institutionalised population, and people with no permanent address were excluded from the study, and younger age groups (15–39 years) were oversampled in order to increase the statistical power in the age group most actively using drugs. Data were collected by Statistics Finland via a self-administered anonymous online/postal questionnaire. The study protocol was approved by the Ethical Review Board of the National Institute for Health and Welfare.

The response rate was 50% (*n* = 3485). Decreasing response rates are an international trend and can be seen in Finland, too. Although the response rate of 50% is tolerable and weighting coefficients were used in order to restore the population representation, a non-response study was also conducted. Statistics Finland collected this data, too. The prevalence of illicit drug use (the main interest of the survey) was found to be similar both among non-respondents and respondents to the original survey. The most common reason (50%) for non-response in the original survey was lack of time, while only 7% did not respond due to the theme of the survey (Karjalainen, Savonen, & Hakkarainen, 2016). Moreover, the prevalence of daily smoking was 14.5%, which is in accordance with other Finnish population-based health studies (Tobacco Statistics 2014, 2015).

### Measurements

The use of e-cigarettes was ascertained via the question “Do you use electronic cigarettes or similar vaporizers?” and the response categories were “Yes, daily or almost daily”; “Yes, occasionally”; “I have used before, but now I have quit”; “I have tried a couple of times”; “No, I have never used”. This question was completed by 3461 respondents. In order to examine the use of e-cigarettes and the determinants associated with it, the question was used to divide the respondents into three mutually exclusive groups. Those who reported using e-cigarettes daily/almost daily or occasionally formed a group called current users (*n* = 70). Ever users were those respondents who said they had tried e-cigarettes a couple of times or used e-cigarettes before, but had since quit (*n* = 416). The third group was never users who reported never having used e-cigarettes (*n* = 2975). The original categories and their distributions are presented in Table 1.

**Table 1.** Use of electronic cigarettes by background variables (%), Finland, 2014.

	**Daily or almost daily (*n* = 25)**	**Occasionally (*n* = 45)**	**Has quit (*n* = 33)**	**Has tried a couple of times (*n* = 383)**	**Never used (*n* = 2975)**	**Total (*n* = 3461)**
**Total**	0.7	1.3	0.9	10.5	86.5	100 (3461)
**Smoking**						
Daily	0.8	5.8	3.4	30.4	59.5	100 (496)
Occasionally	2.0	2.8	2.0	25.4	67.8	100 (354)
Quit	1.4	0.3	0.9	7.7	89.7	100 (989)
Never	0.0	0.2	0.0	2.8	97.0	100 (1616)
**Snus**						
Current	4.3	2.6	6.0	36.8	50.4	100 (117)
Ever	1.8	3.8	2.7	29.5	62.1	100 (599)
Never	0.3	0.7	0.4	5.2	93.4	100 (2723)
**Age**						
15–24 years	1.2	2.7	2.6	25.2	68.3	100 (584)
25–34 years	1.6	2.6	1.5	18.8	75.5	100 (612)
35–44 years	1.2	1.0	0.3	7.7	89.7	100 (584)
45–69 years	0.0	0.5	0.4	3.5	95.6	100 (1687)
**Gender**						
Male	1.0	1.7	1.6	13.5	82.2	100 (1732)
Female	0.4	0.9	0.3	7.5	90.8	100 (1724)
**Education**						
Basic or unknown	1.4	2.5	1.4	15.7	79.1	100 (938)
Intermediate	0.5	1.4	1.1	11.7	85.3	100 (1471)
High	0.4	0.2	0.4	4.3	94.7	100 (1047)
**Marital status**						
Married/co-habiting	0.5	1.1	0.9	7.7	89.8	100 (2107)
Divorced/widowed	0.0	0.8	0.0	6.7	92.5	100 (359)
Single	1.4	2.0	1.5	18.2	77.0	100 (962)
**Employment status**						
Unemployed	2.7	3.8	0.4	14.8	78.4	100 (264)
Student	1.1	2.0	2.2	21.4	73.3	100 (547)
Other	0.1	0.1	0.8	4.2	94.7	100 (758)
Employed/entrepreneur	0.6	1.3	0.6	9.4	88.1	100 (1836)
**Level of urbanisation**						
Urban	0.7	1.4	1.0	11.2	85.6	100 (2636)
Rural	0.9	0.9	0.7	8.4	89.2	100 (802)

In order to examine the prevalence of nicotine-containing liquids in e-cigarettes, the following question was asked: “Have the e-cigarettes you have used contained nicotine?” The response categories were “Always/almost always”; “Sometimes”; “Never”; “I don’t know”.

The use of other tobacco products – cigarettes and snus (Swedish type moist snuff) – were also measured. Smoking was divided into four categories: daily/almost daily, occasionally, have quit, never smoked. The snus users were classified as current users (using daily or occasionally), ever users (has tried a couple of times or used before, but has quit), and never users (has never used snus).

In addition, the analyses made use of the following sociodemographic background factors: respondents’ age (grouped into 15–24, 25–34, 35–44, 45–69 years), gender, education (basic/unknown, intermediate, high), marital status (single, married/co-habiting, divorced/widowed), employment status (employed/entrepreneur, unemployed, student, other), and level of urbanisation (rural, urban).

### Statistical analysis

In order to restore the population representation, differences in response activity and the oversampling of younger age groups were taken into consideration by using weighting coefficients. They were calculated by Statistics Finland and were based on age, gender, education, and level of urbanisation. SPSS Statistics software version 24 was used to analyse the data.

Frequency tables and cross-tabulation were used to describe the data. A multinomial logistic regression model was used to estimate the association between e-cigarette use and different explanatory variables. The use of e-cigarettes (never use/ever use/current use) was an outcome variable, never users being the reference group. Both univariate (Model 1) and adjusted models (Model 2) are presented in Table 2.

**Table 2.** Effects of sociodemographic characteristics and use of other tobacco products on electronic cigarette use, multinomial logistic regression, Finland, 2014.

	**Use of electronic cigarettes**			**Ever use vs. never use**				**Current use vs. never use**			
	Current user %	Ever user %	Never user %	Model 1^A^		Model 2^B^		Model 1^A^		Model 2^B^	
	(*n* = 70)	(*n* = 416)	(*n* = 2975)	*OR*	95% CI	*OR*	95% CI	*OR*	95% CI	OR	95% CI
**Smoking**											
Daily	47.1	42.6	9.9	**19.6**	13.8–27.8	**35.9**	22.9–56.3	**60.6**	18.3– 201.3	**125.3**	28.6–548.8
Occasional	24.3	24.4	8.0	**13.8**	9.5–20.1	**8.5**	5.5–13.2	**37.8**	10.9– 131.5	**33.1**	7.5–146.0
Quit	24.3	21.4	29.7	**3.3**	2.3–4.7	**5.0**	3.2–7.7	**10.3**	2.9– 35.6	**21.3**	4.8–94.1
Never	4.3	11.6	52.4	1.0		1.0		1.0		1.0	
**Snus**											
Current	11.4	12.7	2.0	**14.2**	9.4–21.4	**5.5**	3.2–9.4	**12.6**	5.6– 28.5	**3.9**	1.4–10.9
Ever	48.6	48.9	12.5	**8.7**	6.8–11.0	**3.6**	2.6–4.9	**8.2**	4.9– 13.7	**2.8**	1.5–5.2
Never	40.0	38.5	85.5	1.0		1.0		1.0		1.0	
**Age**											
15–24 years	32.9	40.8	13.3	**10.0**	7.4–13.6	**10.5**	6.1–18.1	**12.0**	5.3– 27.3	**9.4**	3.1–28.2
25–34 years	37.1	31.2	15.5	**6.6**	4.8–9.1	**4.8**	3.2–7.4	**11.6**	5.2– 26.0	**7.9**	3.2–19.6
35–44 years	18.6	11.6	17.5	**2.2**	1.5–3.2	**1.7**	1.1–2.6	**5.1**	2.1– 12.6	**4.0**	1.5–10.3
45–69 years	11.4	16.4	53.7	1.0		1.0		1.0		1.0	
**Gender**											
Male	67.1	66.0	47.6	**2.1**	1.7–2.7	**1.4**	1.0–1.9	**2.3**	1.4– 3.7	1.3	0.7–2.4
Female	32.9	34.0	52.4	1.0		1.0		1.0		1.0	
**Education**											
Basic or unknown	51.4	40.2	24.8	**4.3**	3.1–6.0	1.2	0.8–2.0	**8.0**	3.4– 18.9	**3.7**	1.3–9.9
Intermediate	40.0	47.5	42.0	**3.0**	2.2–4.2	1.2	0.8–1.8	**3.7**	1.5– 8.9	1.8	0.7–4.7
High	8.6	12.4	33.2	1.0		1.0		1.0		1.0	
**Marital status**											
Married/co-habiting	48.6	45.9	63.8	**0.4**	0.3–0.5	0.9	0.6–1.2	**0.4**	0.3– 0.7	0.8	0.4–1.4
Divorced/widowed	4.3	6.1	11.2	**0.3**	0.2–0.4	0.8	0.5–1.5	**0.2**	0.1– 0.6	0.5	0.1–1.9
Single	47.1	48.0	25.0	1.0		1.0		1.0		1.0	
**Employment status**											
Unemployed	23.9	10.2	7.0	**1.7**	1.2–2.4	1.4	0.8–2.2	**3.9**	2.2– 7.1	**2.9**	1.5–5.9
Student	23.9	32.7	13.6	**2.8**	2.2–3.6	**1.6**	1.1–2.5	**2.0**	1.1– 3.5	1.1	0.5–2.5
Other	2.8	10.0	24.4	**0.5**	0.3–0.7	0.9	0.6–1.4	**0.1**	0.0– 0.5	**0.2**	0.0–0.9
Employed/entrepreneur	49.3	47.1	54.9	1.0		1.0		1.0		1.0	
**Level of urbanisation**											
Urban	79.7	81.6	75.9	**1.4**	1.1–1.7	1.1	0.8–1.5	1.2	0.7– 2.2	0.9	0.5–2.1
Rural	20.3	18.4	24.1	1.0		1.0		1.0		1.0	

*Note*. Odds ratios (*OR*) shown in bold type, *p* < 0.05.

^A^Univariate model, includes only one variable at a time.

^B^Adjusted for all the variables shown.

## Results

Of all the respondents, 50.1% were men and the mean age was 43 years (median = 44, *SD* = 16.189). Current e-cigarette users made up 2% of the respondents; 12% were ever users. Of the current users, 77% reported always using nicotine-containing e-liquids, 14% sometimes using, and 9% never using. Ever users used nicotine liquids less often (50% always). All the current e-cigarette users reported knowing whether their e-liquid contained nicotine, but 16% of the ever users reported not knowing whether the e-cigarette they had used contained nicotine or not. The proportion of e-cigarette users always using nicotine was lowest among 15–24 year olds (47%) and highest among 25–34 year olds (65%).

As shown in Table 1, e-cigarette use was most common in the younger age groups: almost one third of those aged 15–24 years and one fourth of those aged 25–34 years had at least experimented with e-cigarettes, while the proportion was just under 5% among the oldest age group. Daily or almost daily use of e-cigarettes was most common among current snus users (4.3%) and the unemployed (2.7%). As many as 40% of daily smokers and half of those currently using snus had at least tried e-cigarettes.

The distribution of demographics and the use of other tobacco products for both current and ever e-cigarette use were rather similar (Table 2), and so were the odds ratios in the univariate models (Model 1). In the adjusted models (Model 2), current or previous tobacco use (cigarettes and snus) increased the odds for both ever and current e-cigarette use compared with never users. Younger age was also associated with both ever and current e-cigarette use. Males had higher odds for ever e-cigarette use (*OR* = 1.4, 95% CI 1.0–1.9), but there were no gender differences among current users. In terms of employment status, being a student was associated with ever use, whereas unemployment was associated with current e-cigarette use. No statistically significant difference in education was found for ever users in the adjusted model, but lower education was associated with current e-cigarette use when compared to those with higher education (*OR* = 3.7, 95% CI 1.3–9.9). The differences in marital status or level of urbanisation did not reach statistical significance in the adjusted models.

## Discussion

In 2014, the use of e-cigarettes was rather low among the general Finnish population: 2% of Finnish adults reported using e-cigarettes currently and 12% reported having ever used them. The use of tobacco products and younger age were strongly associated with both forms of e-cigarette use. Unemployment and lower education were associated with current e-cigarette use, and being a student was associated with ever use of e-cigarettes.

Current use of e-cigarettes seems to be at roughly similar levels in Finland and the other Nordic countries, although some variation exists (European Commission, 2015). This may be explained by different regulatory environments, which seem to be associated with e-cigarette use (Yong et al., 2015). However, e-cigarette regulation is always nested within other national tobacco control policies and the stage of the tobacco epidemic (Thun, Peto, Boreham, & Lopez, 2012) in general. This makes it difficult to draw direct conclusions of the associations between e-cigarette regulation and the prevalence of use in different countries.

As shown by earlier research (Farsalinos, Poulas, Voudris, & Le Houezec, 2016), smoking is strongly associated with e-cigarette use. In our study, snus use was also associated with e-cigarette use, similarly to earlier results among Finnish adults and adolescents (Kinnunen et al., 2015; Ruokolainen et al., 2016) as well as among Swedish adolescents (Geidne, Beckman, Edvardsson, & Hulldin, 2016). Given the cross-sectional nature of our data, we were not able to study the trajectories in the use of different tobacco products and e-cigarettes, and the interplay of different products in regular or occasional use. In general, this is an area of research where more longitudinal studies are needed. In addition, qualitative and mixed-methods studies could bring important insights into the use of multiple tobacco or nicotine products. The current evidence is inconclusive. Most studies indicate e-cigarette use occurs primarily among smokers (Glasser et al., 2017), but some have found e-cigarette use to occur among never-smokers and even predict later use of combustible tobacco in young populations (US Department of Health and Human Services [USDHHS], 2016).

As far as the authors know, prior Nordic studies of the demographic determinants of e-cigarette use in the adult population have not been published (excluding Ruokolainen et al., 2016, in Finnish). Our finding that the ever use of e-cigarettes was associated with being a student was similar to earlier research conducted in the European Union member states (Ooms, Bosdriesz, Portrait, & Kunst, 2016). This, together with the association with younger age, and the common use of non-nicotine e-liquids in the youngest age group, may indicate some curiosity behind e-cigarette use in younger age groups, as commonly reported elsewhere (USDHHS, 2016).

The finding that unemployment and lower education were associated with current but not ever use of e-cigarettes may indicate that individuals who could be having financial difficulties are trying to seek cheaper substitutes for cigarettes. At the time of our data collection, e-cigarettes were not taxed, and they may therefore have been cheaper to use than conventional cigarettes, depending for example on the volume and patterns of use. However, the taxation of e-cigarettes and e-liquids was enacted as of 2017, warranting further monitoring of its effects in different socioeconomic groups. Another explanation, although less likely according to the previous findings (Hiscock et al., 2012 ), is that the unemployed and people with lower education levels are more likely than other groups to be trying to quit smoking. Also, their attempts to quit may have been unsuccessful and they might try to switch from conventional cigarettes to e-cigarettes for harm-reduction purposes. A previous study found no differences between e-cigarette use as a cessation tool among different socioeconomic groups (Ooms et al., 2016), but another study has suggested that those facing financial difficulties may be more likely to have experimented with e-cigarettes as cessation aids (Filippidis, Laverty, & Vardavas, 2016). Unfortunately, our data did not provide measures related to smoking cessation. Future e-cigarette research should look into their role in smoking cessation among different socioeconomic groups.

While e-liquids containing nicotine were available only from international online retailers or as having been brought from travels at the time of the data collection, most current e-cigarette users reported always using nicotine e-liquids. Hence, the previous regulatory environment enabled access to nicotine e-liquids for those interested in their use, with limited availability and visibility for others. The new regulatory environment poses many important questions for research. Will the new regulations (say, the prohibition on distance sales or the ban on characterising flavours) have an impact on the e-cigarette use in the general population, or among different subgroups, such as current smokers or those in different socioeconomic groups? Will the better availability have an impact on the impulse purchases of nicotine e-cigarettes among quitters or recent ex-smokers? Will retail outlets be able to prevent the sales of nicotine e-cigarettes or liquids to minors?

These questions are important also for policy-makers, as Finland aims to be both tobacco and nicotine free by the year 2030. Therefore, the impact of the new regulations needs to be monitored at the population level. A specific group of interest consists of current smokers and recent quitters. In the Finnish tobacco control policy, smoking cessation services are among the development targets (Joossens & Raw, 2017). New nicotine-containing products on the market might make the situation more complex, as health professionals have to take stands on safety and the role of e-cigarettes in smoking cessation in the absence of robust evidence. The demand for cessation services may also fluctuate increasingly due to smokers switching to continued e-cigarette use or dual use instead of trying to quit smoking or nicotine use completely (see Manzoli et al., 2016). As this study includes the normal limitations of cross-sectional studies, no causal inferences can be drawn, for example, on whether e-cigarette use preceded smoking or the other way around. Longitudinal data are needed to answer these questions.

## Conclusions

In the former regulatory environment, prior to the 2016 renewed Tobacco Control Act, current e-cigarette use in the Finnish adult population was rather low. E-cigarette use was more likely among tobacco users, younger adults, students, and respondents with lower education. The impact of the new policy needs to be monitored closely, as nicotine-containing e-cigarettes and e-liquids can now enter the national market, with only retailer licensing instead of the formerly required medicinal marketing authorisation. As Finland aims to be both tobacco and nicotine free by 2030, youth access to e-cigarettes must be prevented and the existing cessation services need to be developed to support quitting of both smoking and e-cigarette use.
